# Supplementary material for: Berberine Regulation of Cellular Oxidative Stress, Apoptosis and Autophagy by Modulation of m6A mRNA Methylation through Targeting the Camk1db/ERK Pathway in Zebrafish-Hepatocytes
Source: Antioxidants (Basel). 2022 Nov 30;11(12):2370. doi: 10.3390/antiox11122370 (PMC9774189; doi:10.3390/antiox11122370)

Supporting Information

Title： Berberine Regulation of Cellular Oxidative Stress, Apoptosis and Autophagy by Modulation of m^{6}A mRNA Methylation through Targeting the *Camk1db*/ERK Pathway in Zebrafish-Hepatocytes

Meijuan Zhang [1], Jin Liu [1], Chengbing Yu [1], Shangshang Tang [1], Guangzhen Jiang [2], Jing Zhang [1], Hongcai Zhang [1], Jianxiong Xu [1] and Weina Xu [1,*]

[1] Shanghai Key Laboratory for Veterinary and Biotechnology, School of Agriculture and Biology, Shanghai Jiao Tong University, No. 800 Jiangchuan Road, Shanghai 200240, China

[2] Key Laboratory of Aquatic Nutrition and Feed Science of Jiangsu Province, College of Animal Science and Technology, Nanjing Agricultural University, No. 1 Weigang Road, Nanjing 210095, China

* Correspondence: xuweina@sjtu.edu.cn

The file includes 6 tables, 2 figures.

Table S1 Distribution of m6A peaks.

Table S2 Statistics of the number of differentially methylated genes.

Table S3 Peaks distribution statistics of differentially methylated genes.

Table S4 Transcription level change statistics.

Table S5 Statistics of changes in methylation level and transcription level.

Table S6 Changes in the methylation level and transcription level of Camk1db gene.

Figure S1 ROS fluorescence microscope picture. The red arrow is the fluorescence of ROS in zebrafish liver cells. The higher the ROS content, the stronger the fluorescence intensity. Date was presented as mean ± SEM (n=3).

Figure S2 Flow cytometry detection graphs. The cell count in each group is 10,000.

Table S1

Table S1. Distribution of m^{6}A peaks

Group	5'UTR (%)	3'UTR (%)	1st Exon (%)	Other Exon (%)
Control	25.12	43.3	6.43	25.16
SP	25.55	45.6	5.88	22.97
BBR	31.22	37.69	6.66	24.43
SP+BBR	31.22	36.36	7.04	25.39

Table S2

Table S2. Statistics of the number of differentially methylated genes

Comparison Group	Number of genes upregulated by methylation *	Number of genes down-regulated by methylation *
SP / Con	135	96
BBR / Con	300	188
SP+BBR / Con	467	331
BBR / SP	243	250
SP+BBR / SP	65	42
SP+BBR / BBR	254	187

Note: Differential genes were screened by fold-change and p-value of univariate analysis. Screening conditions: 1) fold-change ≥ 2; 2) p-value < 0.05, the intersection of the two, and the common genes, namely differential genes, were obtained. * $p < 0.05$, $fc \geq 2$.

Table S3

Table S3. Peaks distribution statistics of differentially methylated genes

Comparison Group	5'UTR (%)	3'UTR (%)	1st Exon (%)	Other Exon (%)
SP / Con	25.91	46.03	6.09	21.97
BBR / Con	32.37	41.46	5.91	20.26
SP+BBR / Con	29.35	45.42	5.31	19.92
BBR / SP	27.43	48.24	5.95	18.38
SP+BBR / SP	26.39	49.77	5.06	18.79
SP+BBR / BBR	33.28	37.23	6.32	23.17

Table S4

Table S4. Transcription level change statistics

Comparison Group	Number of transcript expression upregulation *	Number of transcript expression down-regulation *
SP / Con	208	195
BBR / Con	1468	754
SP+BBR / Con	1539	1037
BBR / SP	1220	654
SP+BBR / SP	1258	789
SP+BBR / BBR	254	413

Note: Differential genes were screened by fold-change and p-value of univariate analysis. Screening conditions: 1) fold-change ≥ 2; 2) p-value < 0.05, the intersection of the two, and the common genes, namely differential genes, were obtained. * $p < 0.05$, $fc \geq 2$.

Table S5

Table S5. Statistics of changes in methylation level and transcription level

Comparison Group	Number of methylation up/down-regulated	Number of transcripts up/down-regulated	Number of significant common differences
SP / Con	135 / 96	176 / 171	2
BBR / Con	300 / 188	1456 / 1387	64
SP+BBR / Con	467 / 331	1464 / 983	100
BBR / SP	243 / 250	1135 / 603	49
SP+BBR / SP	65 / 42	1173 / 738	106
SP+BBR / BBR	254 / 187	13 / 50	6

Table S6

Table S6. Changes in the methylation level and transcription level of Camk1db gene

Comparison Group	Methylation level	Transcript level
SP / Control	Down *	Down
BBR / SP	Up *	Up *
BBR+SP / SP	Up *	Up *

Note: Differential genes were screened by fold-change and p-value of univariate analysis. Screening conditions: 1) fold-change ≥ 2; 2) p-value < 0.05, the intersection of the two, and the common genes, namely differential genes, were obtained. * $p < 0.05$, $fc \geq 2$.

Figure S1

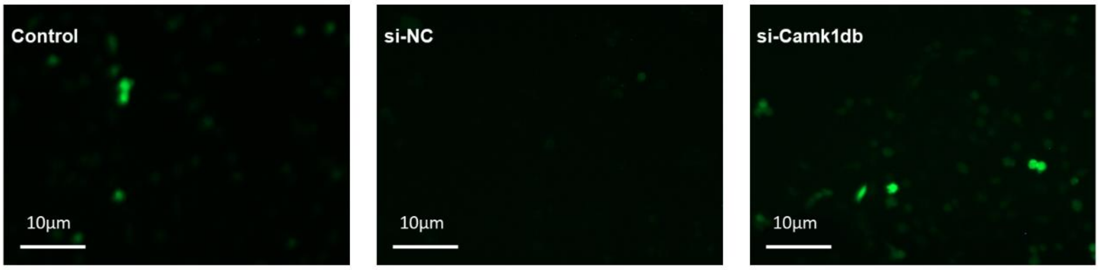

Figure S2

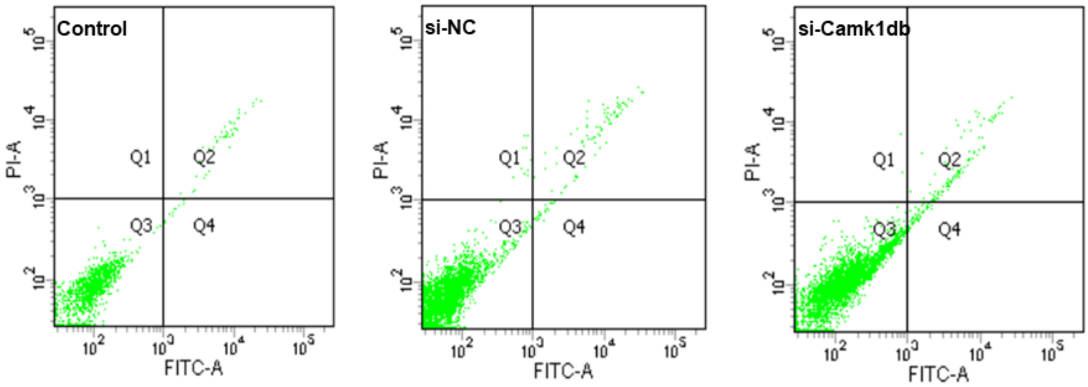

Supplement: Supplementary file 1 [file antioxidants-11-02370-s001.zip › antioxidants-1973474-supplementary.pdf]
